# Individual and Facility-Level Determinants of Iron and Folic Acid Receipt and Adequate Consumption among Pregnant Women in Rural Bihar, India

**DOI:** 10.1371/journal.pone.0120404

**Published:** 2015-03-20

**Authors:** Amanda Wendt, Rob Stephenson, Melissa Young, Amy Webb-Girard, Carol Hogue, Usha Ramakrishnan, Reynaldo Martorell

**Affiliations:** 1 Nutrition and Health Sciences, Division of Biological and Biomedical Sciences, Emory University, Atlanta, Georgia, United States of America; 2 Epidemiology and Biostatistics Unit, Institute of Public Health, Heidelberg University, Heidelberg, Germany; 3 Hubert Department of Global Health, Rollins School of Public Health, Emory University, Atlanta, Georgia, United States of America; 4 Department of Epidemiology, Rollins School of Public Health, Emory University, Atlanta, Georgia, United States of America; Harvard School of Public Health, UNITED STATES

## Abstract

**Background:**

In Bihar, India, high maternal anemia prevalence and low iron and folic acid supplement (IFA) receipt and consumption have continued over time despite universal IFA distribution and counseling during pregnancy.

**Purpose:**

To examine individual and facility-level determinants of IFA receipt and consumption among pregnant women in rural Bihar, India.

**Methods:**

Using District Level Household Survey (2007–08) data, multilevel modeling was conducted to examine the determinants of two outcomes: IFA receipt (any IFA receipt vs. none) and IFA consumption (≥90 days vs. <90 days). Individual-level and facility-level factors were included. Factor analysis was utilized to construct antenatal care (ANC) quality and health sub-center (HSC) capacity variables.

**Results:**

Overall, 37% of women received any IFA during their last pregnancy. Of those, 24% consumed IFA for 90 or more days. Women were more likely to receive any IFA when they received additional ANC services and counseling, and attended ANC earlier and more frequently. Significant interactions were found between ANC quality factors (odds ratio (OR): 0.37, 95% confidence interval (CI): 0.25, 0.56) and between ANC services and ANC timing and frequency (OR: 0.68, 95% CI: 0.56, 0.82). No HSC factors were significantly associated with IFA receipt. Women were more likely to consume IFA for ≥90 days if they attended at least 4 ANC check-ups and received more ANC services. IFA supply at the HSC (OR: 1.37, 95% CI: 1.04, 1.82) was also significantly associated with IFA consumption.

**Conclusions:**

Our findings indicate that individual and ANC factors (timing, frequency, and quality) play a key role in facilitating IFA receipt and consumption. Although HSC capacity factors were not found to influence our outcomes, significant variation at the facility level indicates unmeasured factors that could be important to address in future interventions.

## Introduction

The World Health Organization (WHO) estimates that 56 million pregnant women (42%) globally are anemic[1], the majority of whom live in resource poor settings[2]. The largest number of individuals affected (18.1 million) live in South-East Asia[1]. In Bihar, India, the anemia prevalence is even higher, affecting 60% of pregnant women in one of the largest and poorest states of the country[3].

During pregnancy, anemia can lead to several adverse outcomes including low birth weight, preterm delivery, stillbirth, and maternal and neonatal mortality[4–6]. Over half of anemia cases are estimated to be due to iron deficiency[2]. Fortunately, an efficacious and cost-effective strategy exists for iron deficiency anemia prevention and control. Daily oral iron supplementation success has been well documented in both hematological improvements[7] and clinical outcomes such as increased birth weight[8].

Since 1970, the Government of India has had an iron supplementation program, which expanded to provide universal iron supplementation for pregnant women in 1992[9, 10]. However, evaluations, conducted from 1988–2007, have shown a lack of success both in implementation and outcomes[11–15]. Anemia prevalence among pregnant women in Bihar has increased from 46.4% in 1998–9 to 60.2% in 2005–06[16]. In addition, recent evaluations of iron and folic acid supplement (IFA) consumption show only a low number of women receiving any IFA or consuming an adequate amount during pregnancy. In Bihar, National Family Health Survey, Round 3 (NFHS-3) (2005–06) data show 29.7% received IFA and only 9.7% of women consumed it for 90 or more days during their last pregnancy[3]. In 2009, UNICEF reported that only 6.7% of Bihari women consumed 100 or more IFA (tablets or syrup) and 67.3% had consumed none at all[17].

Government provided IFA is distributed through antenatal care (ANC), which in rural areas is provided at health sub-center (HSC) facilities. As the most peripheral health facility, the HSC serves as the initial point of contact between the community and health system. Due to this role, “the success of any nation-wide program would depend largely on the well-functioning HSCs providing services of acceptable standard to the people”[18]. Therefore, a woman’s receipt and consumption of IFA does not solely depend on individual and household level factors, but is also shaped by the capacity and service quality of her HSC facility.

Though several studies have examined individual factors associated with IFA receipt or adherence, few have addressed the role of ANC quality or facility capacity[19–23]. To our knowledge, none have accounted for facility characteristics as contextual factors by conducting multi-level modeling, which may show the impact that facilities make to women’s IFA receipt and consumption beyond individual-level factors alone.

Thus our objective was to more robustly examine individual and facility-level determinants of IFA receipt and consumption among pregnant women in rural Bihar. We used data from the District Level Household Survey—Round 3 (2007–08), a nationally representative survey which recorded women’s reported IFA receipt and consumption, ANC attendance and quality, and facility-level characteristics[24] and employed a multi-level hierarchical logistic regression to account for variance at the facility level.

## Methods

### Data Sources

We analyzed data from the third round of the District Level Household Survey (DLHS-3), completed in 2007–08, from the state of Bihar. DLHS-3 is a cross-sectional survey that provides representative data at national, state, and district levels by using a multi-stage stratified probability proportion to size sampling design[24]. Purposes of this survey included measuring maternal and child healthcare utilization as well as health facility capacity and effectiveness[24]. In Bihar, 46,840 ever-married women (15–48y) were surveyed. Household (n = 47,137) and village (n = 1,668) questionnaires were also conducted with household members and village representatives, respectively. Facilities surveyed included HSCs (n = 1,165), Primary Health Centers (n = 524), and Community Health Centers (n = 66). The response rates in Bihar for ever-married women and HSCs were 86.7% and 92.8%, respectively. HSC response rate was calculated using individual district reports[25].

This was a secondary data analysis conducted on de-identified and publicly available data; as such it did not require approval by an institutional review board.

### Inclusion & Exclusion Criteria

For this analysis, we used data from ever-married women, village, and HSC surveys. For our two outcomes, (IFA receipt and IFA consumption), we constructed two final samples.

**Fig 1:**
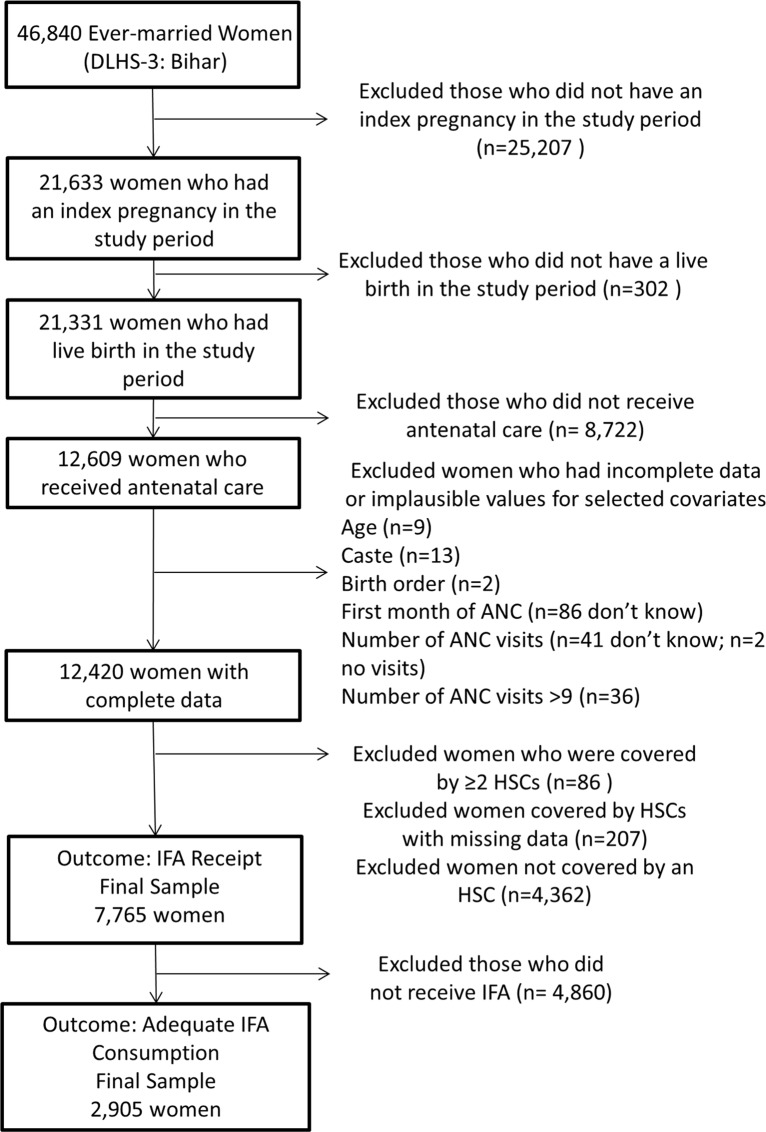
Flowchart of ever-married women exclusions and final sample. Flowchart of exclusions and final sample of ever-married women surveyed in Bihar through DLHS-3. DLHS: District Household Survey; ANC: Antenatal care; HSC: Health Sub-Center; IFA: iron and folic acid tablets or syrup.

We included women in our data analysis that had a live birth in the time period surveyed (from January 1, 2004 to 2007–08), lived in a primary sampling unit (PSU) covered by only one HSC, and had complete data for the outcome of interest and covariates. Due to these inclusion criteria, all women who did not attend ANC (and thus were not asked about IFA receipt or consumption) and those who lived in urban residences (and were not covered by an HSC) were excluded. Women with incomplete or implausible data were also excluded. In our IFA consumption model, women who did not receive IFA were not asked about consumption and therefore excluded from that model (Fig. 1).

**Fig 2:**
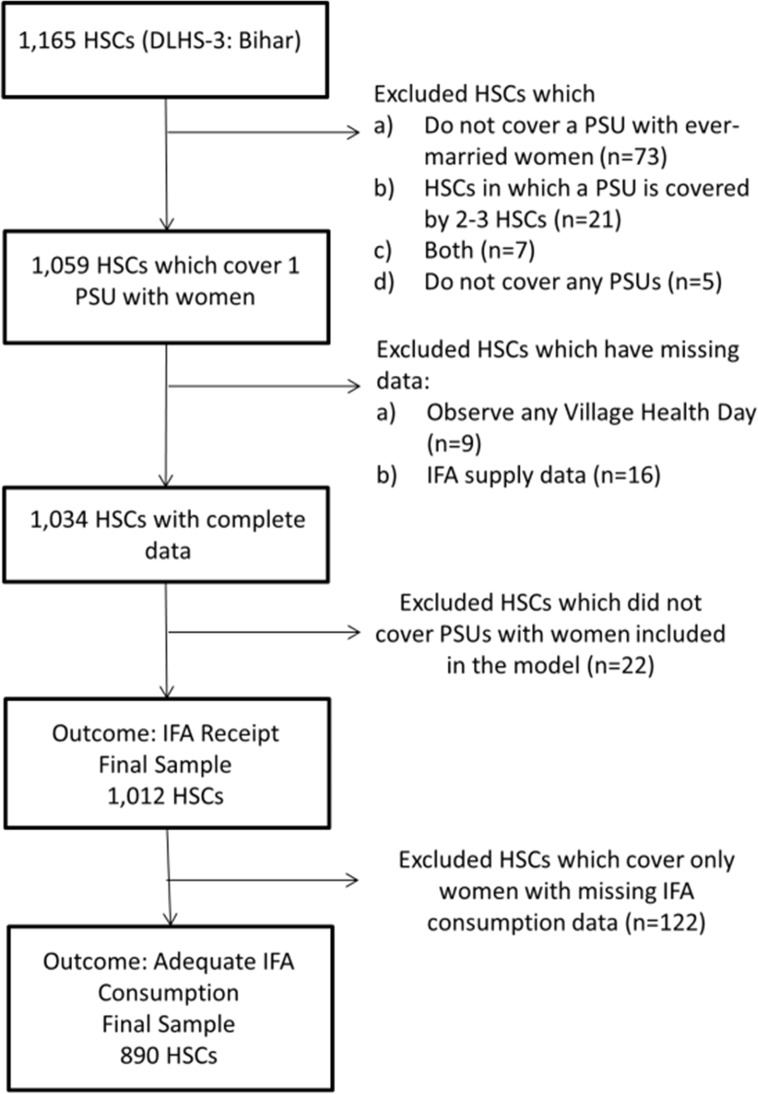
Flowchart of Health Sub-Center exclusions and final sample. Flowchart of exclusions and final sample of health sub-centers surveyed in Bihar through DLHS-3. HSC: Health Sub-Center; DLHS: District Level Household Survey; PSU: Primary Sampling Unit; IFA: iron and folic acid tablets or syrup.

We also excluded HSCs that did not cover a primary sampling unit (PSU) with an ever-married woman in our model, that covered more than one PSU, or that had missing data. In rural populations, PSUs were defined as census villages or communities[24]. For the IFA receipt outcome, we included 1,012 HSCs and for the IFA consumption outcome, 890 HSCs (Fig. 2).

There were some differences between the total sample and our analysis samples. Women included in our models were older, more educated, of higher birth order, and less likely to be married before the age of 18 or be of a scheduled caste or tribe. Women included in our second model (IFA consumption), were more likely to have initiated ANC earlier, have more frequent ANC visits, and experienced more ANC practice and counseling. HSCs with poor infrastructure were more likely to be excluded from the analysis.

### Dependent Variables

We constructed two primary outcomes for this analysis. Both outcomes were dichotomous variables regarding IFA supplements provided during ANC as routine standard of care through the government health system. Government guidelines require one daily dose of IFA to be 100 mg elemental iron (ferrous sulphate[18]) and 500 mcg folic acid from tablets or syrup[26].

The first outcome was defined as receipt of any iron and folic acid supplements during the last pregnancy. Interviewers asked women who reported having attended ANC how many IFA tablets or bottles they received during their last pregnancy. This included any quantity of IFA tablets or IFA syrup. Those who answered any number greater than zero were classified as having received IFA [25].

Women who received IFA were asked how many days they consumed IFA tablets / syrup during their last pregnancy occurring after January 1, 2004[25]. The second outcome was whether the woman reported consuming IFA tablets or syrup for 90 days or more and was generated only for women who reported that they received IFA during their last pregnancy.

### Independent Variables

#### ANC

ANC variables included receipt and quality measures. ANC receipt was measured by a timing variable (Early enrollment (1^st^ trimester) vs. late enrollment (2^nd^ or 3^rd^ trimester) of first ANC visit) and a frequency variable (<4 ANC visits vs. ≥4 ANC visits) according to WHO standards[27]. ANC quality was comprised of 18 practices and counseling topics measured by the survey instrument. These topics did not address IFA administration or counseling but were included as an overall quality measure. Nine covered ANC practices (weight, height, blood pressure, blood test, urine test, breast exam, abdomen exam, sonogram/ultrasound, and delivery date given) and nine reviewed ANC counseling topics (advice regarding delivery, nutrition, breastfeeding, keeping the baby warm, cleanliness at delivery, family planning for spacing and limiting, improved maternal and child nutrition, and importance of institutional delivery). We removed 5 variables (blood pressure, urine test, delivery advice, keeping the baby warm, and family planning for limiting) from the analysis because they were too highly correlated, causing a singular matrix. We used the remaining variables to conduct an exploratory factor analysis using polychoric correlation matrices. These have been shown to result in more accurate correlations between categorical variables[28]. We then extracted factors from a principal components analysis and rotated them orthogonally using the varimax method. The 13 variables separated into two distinct factors with eigenvalues >1: ANC practice and ANC counseling topics. We retained variables in factors which contained factor loadings of ≥ 0.5. These two factors explained 89% of the total variance (Table 1).

**Table 1:** Characteristics and rotated factor loadings for antenatal care factors^a^ ^a^.

	ANC Practices	ANC Counseling
Eigenvalue	7.16	1.60
Proportion variance explained	0.73	0.16
*Rotated factor loadings*		
Blood tested	0.87	-
Weight measured	0.81	-
Abdomen examined	0.81	-
Sonogram/ultrasound taken	0.80	-
Breast examined	0.76	-
Height measured	0.76	-
Delivery date given	0.72	-
Importance of cleanliness at delivery	-	0.83
Better nutrition for mother and child	-	0.80
Family planning for spacing	-	0.75
Breastfeeding	-	0.75
Importance of institutional delivery	-	0.73
Nutrition advice	-	0.59

^a^ Factor loadings ≤|0.5| are not shown; ANC: Antenatal care

#### HSC

We conducted a factor analysis on 12 HSC characteristics. Three of these were not included in the final factors because they had low loading values on all factors. Excluded variables consisted of the ratio of pregnant women registered in the previous month to HSC coverage population, sufficient printed ANC cards, and an Auxiliary Nurse Midwife (ANM) residing in the HSC village. The other 9 variables formed three factors: Village Health Day and Primary Health Center (PHC)/village monitoring, Personnel characteristics, and HSC infrastructure. The first factor, Village Health Day and PHC/village monitoring, is comprised of the observation of Village Health Day, and PHC or village monitoring activities. A large part of the PHC and Village Health and Sanitation Committee (VHSC) activities center around monitoring progress of the HSC’s Village Health Day, at which IFA is distributed to pregnant women among other ANC activities. The second factor, Personnel characteristics, includes the number of HSC personnel, number of training topics attended in the past five years, and receipt and utilization of untied funds. These characteristics may influence ANC coverage of the village, capacity of the workers to distribute and counsel on IFA benefits and consequences, and may indicate more active HSC workers. The third factor, Sub-Center Infrastructure, reflects structural capacity. This factor is represented by building condition (as interpreted by the interviewer to be satisfactory or in need of repair[29]), and access to water. We conducted polychoric factor analysis on these factors as described above. The three factors selected had eigenvalues of 1.10, 0.63, and 0.54. The 3 factors together explained 87% of the cumulative variance (Table 2).

**Table 2:** Characteristics and rotated factor loadings for Health Sub-Center factors^a^ ^a^.

	Village Health Day & PHC/Village Monitoring	Personnel Characteristics	Sub-Center Infrastructure
Eigenvalue	1.10	0.63	0.54
Proportion variance explained	0.42	0.24	0.21
*Rotated factor loadings*			
Observation of any Village Health Day	0.56	-	-
Written feedback from PHC	0.50	-	-
VHSC present in some villages in HSC area	0.42	-	-
Medical Officer visited HSC in previous month	0.36	-	-
Received and utilized untied funds from previous financial year	-	0.37	-
HSC personnel	-	0.46	-
HSC training	-	0.44	-
Present condition of existing building	-	-	0.44
Water available at Sub-Center	-	-	0.43

^a^ Factor loadings ≤|0.35| are not shown;

HSC: Health Sub-Center; PHC: Primary Health Center; VHSC: Village Health and Sanitation Committee

### Covariates

Individual-level covariates also included in the model have each been shown to be associated with anemia, maternal health service utilization, or IFA receipt. These were maternal age at index birth[30, 31], age of marriage[31, 32], maternal education[33, 34], gender composition of living children[35], birth order of index pregnancy[30, 33, 36], caste[19], religion[19], and household wealth quintile[19] (Table 3).

**Table 3:** Variable definitions included in multilevel modeling of IFA receipt and consumption.

*Variables*	*Description*
*Facility-level variables*	
HSC factors	Estimated factor scores calculated to describe overall HSC capacity
HSC factor: Village Health Day and PHC/village monitoring	Estimated factor score describing Village Health Day observation and PHC/village support and monitoring
Observation of any Village Health Day	If the HSC worker conducts Village Health Day: a grouping of activities which includes IFA distribution to pregnant women—No (Ref.); Yes
Any written feedback from the PHC	If any written feedback from the PHC has been received in the previous month—No (Ref.); Yes
Village Health and Sanitation Committee	If a Village Health and Sanitation Committee is present in some or all of the villages in the HSC coverage area—No (Ref.); Yes
Medical Officer visit	If a Medical Officer has visited the HSC in the previous month—No (Ref.); Yes
HSC factor: Personnel characteristics	Estimated factor score describing personnel characteristics
Untied funds	If HSC untied funds were received and at least partially utilized in the previous year—No (Ref.); Yes
Number of personnel	Number of health workers at the HSC at the time of survey—0–1 workers (Ref.); ≥2 workers
Personnel training	Number of training topics received by HSC workers in the last 5 years—0–3 topics (Ref.); 4–8 topics
HSC factor: Sub-Center infrastructure	Estimated factor score describing HSC infrastructure quality
Present condition of building	Present condition of HSC building as observed by interviewer—Needs repair (Ref.); Satisfactory
Water available at Sub-Center	If there is a source of water at the HSC—No (Ref.); Yes
IFA stock available on day of survey	If IFA is available on the day of the survey—No (Ref.); Yes
Distance to nearest HSC	Distance in km from the village to the nearest HSC—In village (Ref.); <5 km away; ≥5 km away
*Individual-level variables*	
Age	Maternal age at index birth—<20 y; 20–24 y; >24 y (Ref.)
Age at marriage	Maternal age at marriage—<18 y; ≥18 y (Ref.)
Maternal education	Highest level of education attained in years—None or Don’t know (Ref.); 1–4 y; 5–8 y; ≥9 y
Gender composition of living children	Presence of living sons—No (Ref.); Yes
Birth order of index pregnancy	Birth order of the index pregnancy—1^st^ birth; 2^nd^ birth; 3^rd^ or greater birth (Ref.)
Caste	Caste of woman—scheduled caste or tribe; others (Ref.)
Religion	Religion of woman—Hindu (Ref.); Muslim or other
Household Wealth Index quintiles	Index of household assets calculated at the national level and divided into quintiles[25]—Poorest (Ref.); Second; Middle; Fourth; Richest
Husband’s education	Highest level of education attained by husband in years—None or Don’t know (Ref.); 1–4 y; 5–8 y; 9–12 y; ≥12 y
ANC timing and frequency	Recommended frequency and Initiation of ANC according to WHO standards[27]—Early enrollment^a^ and ≥4 visits; Late enrollment^a^ and ≥4 visits; Early enrollment^a^ and <4 visits; Late enrollment^a^ and <4 visits (Ref.)
ANC factors	Estimated factor scores calculated to describe overall ANC quality
ANC factor: Practices	Estimated factor score describing specific practices occurring during ANC visits.
Abdomen examined	Components of ANC visits identified by women’s self-report[25].—No (Ref.); Yes
Blood tested	
Breast exam	
Delivery date given	
Height measured	
Sonogram/ultrasound taken	
Weight measured	
ANC factor: Counseling	Estimated factor score describing specific counseling topics covered during ANC visits.
Better nutrition for mother and child	Counseling topics addressed during ANC visits identified by women’s self-report[25].—No (Ref.); Yes
Breastfeeding	
Cleanliness at delivery	
Family planning—spacing	
Importance of institutional delivery	
Nutrition advice	

^a^ Early enrollment:

1^st^ trimester, Late enrollment: 2^nd^–3^rd^ trimester; ANC: Antenatal Care; HSC: Health Sub-Center; IFA: Iron and Folic Acid; PHC: Primary Health Center; PSU: Primary Sampling Unit; Ref.: Reference Value; WHO: World Health Organization

### Statistical Analyses

We conducted descriptive statistics, examining frequencies and percentages. We also reported the proportion of women who received any IFA and who consumed IFA for 90 or more days during their last pregnancy by each covariate along with chi-square tests of significance. All descriptive statistics and modeling took into account weighting and clustering at the primary sample unit level.

Data cleaning and factor analyses were completed in SAS 9.3 (SAS Inc., Cary, NC, USA). We assessed collinearity using the COLLIN macro and accounted for clustering using PROC SURVEYLOGISTIC[37] in SAS 9.3.

We accounted for the hierarchical nature of the data by conducting a multilevel logistic model. We included 2 levels: individual and facility (primary sampling unit). To assess the need for an additional level (household), we put household identifiers in the model. This was not significant nor did it change our estimates so we present the 2-level model here, using the *xtlogit* command in Stata version 13 (StataCorp, College Station, TX, USA). We also made an *a priori* decision to assess possible interactions between all ANC variables. This was because women who received more ANC services may have been more likely to receive more counseling and attend ANC more frequently or earlier. For each outcome variable, six models were constructed, each including a set of factors: 1) Individual Factors, 2) Individual and ANC Timing and Frequency factors, 3) Individual and ANC Quality Factors, 4) Individual and HSC Factors, 5) All Factors, and 6) All Factors including Interactions. For each model, we used Akaike Information Criterion (AIC) as a measure of goodness-of-fit in comparing final models[38]. We also report the facility-level random effects. These measure the extent to which the outcome varies by PSU, while controlling for all other covariates. This can signal residual variation due to measures not included in the model or not measured by this survey, such as differences in beliefs or social norms surrounding iron consumption in communities, or facility variations that were not measured here.

## Results

### Sample Characteristics

In our IFA receipt model, our final sample consisted of 7,765 women and 1,012 HSCs. For the outcome IFA consumption, our final sample included 2,905 women and 890 HSCs.

Overall, 37.4% of women received any IFA during their last pregnancy. Of those, 23.8% consumed IFA for at least 90 days. Women included in the IFA receipt model were predominantly 20 years or older (84.1%), married before the age of 18 (72.1%), uneducated (62.0%), Hindu (83.5%), more likely to be in the two lowest wealth quintiles (67.2%), have one or more sons (77.1%), and have a birth order of three or greater (51.8%). Although all women received at least one ANC check-up, 50.3% did not receive any ANC practices and 36.6% did not receive any counseling messages that were surveyed (Table 4). Most women included in our sample had an HSC in her village (40.0%) or were located within 5 km of an HSC (46.0%). Other public health facilities (e.g. Primary Health Centers, District Hospitals) were less likely to be available in or within 5 km (5.6% and 26.9%, respectively; *data not shown*).

**Table 4:** Individual-level factors of study population by prevalence of iron and folic acid receipt and consumption.

			*Received any IFA*	*Chi-Square*		*Consumed IFA for ≥90 days*	*Chi-Square*
		N	%	p-value	N	%	p-value
**Overall**		7765	37.4		2905	23.8	
**Age**							
	*<20 y*	1237	37.8	<0.0001	1148	22.4	0.2352
	*20–24 y*	3071	42.0		468	23.1	
	*>24 y*	3457	33.2		1289	25.2	
**Age of marriage**							
	*<18 y*	5602	33.2	<0.0001	1859	20.3	<0.0001
	*≥18 y*	2163	48.3		1046	29.8	
**Mother's education**							
	*None / Don't know*	4812	28.7	<0.0001	1382	14.6	<0.0001
	*1–4 y*	610	36.7		224	18.3	
	*5–8 y*	1202	46.5		559	25.9	
	≥*9 y*	1141	64.9		740	40.8	
**Gender composition of living children**							
	*No sons*	1782	41.2	0.0002	2171	22.9	0.0591
	*≥1 sons*	5983	36.3		734	26.3	
**Birth order of index pregnancy**							
	*1*	1981	45.5	<0.0001	1251	19.0	<0.0001
	*2*	1758	42.8		901	29.3	
	*≥3*	4026	31.1		753	25.0	
**Caste**							
	*Scheduled castes or tribes*	1671	33.8	0.0017	565	14.5	<0.0001
	*Others*	6094	38.4		2340	26.0	
**Religion**							
	*Hindu*	6486	38.9	<0.0001	2527	24.3	0.1135
	*Muslim & others*	1279	29.6		378	20.4	
**Wealth Index quintiles**							
	*Poorest*	2154	26.5	<0.0001	571	13.0	<0.0001
	*Second*	3061	33.4		1023	19.3	
	*Middle*	1510	44.4		671	23.3	
	*Fourth*	835	59.0		493	37.8	
	*Richest*	205	71.7		147	52.3	
**Husband's education**							
	*None / Don't know*	2714	25.9	<0.0001	702	12.8	<0.0001
	*1–4 y*	700	33.1		232	14.2	
	*5–8 y*	1553	35.8		556	18.4	
	*9–12 y*	2315	47.9		1109	30.4	
	*>12 y*	483	63.3		306	41.8	
**ANC timing & frequency** ^**a**^				<0.0001			<0.0001
	*Early enrollment* ^*b*^ *& ≥4 ANC Visits*	837	71.7		600	49.5	
	*Late enrollment* ^*b*^ *& ≥4 ANC Visits*	215	63.7		137	40.9	
	*Early enrollment* ^*b*^ *& <4 ANC Visits*	2232	40.3		900	17.6	
	*Late enrollment* ^*b*^ *& <4 ANC Visits*	4481	28.3		1268	14.1	
**ANC Practices** ^**a**^				<0.0001			<0.0001
	*None*	3903	19.2		749	9.2	
	*1–3*	2630	48.9		1287	19.6	
	*4–7*	1232	70.5		869	42.4	
**ANC Counseling** ^**a**^				<0.0001			<0.0001
	*None*	2841	21.3		605	15.4	
	*1–3*	3376	41.0		1385	20.4	
	*4–7*	1548	59.1		915	34.3	

^a^ It should be noted that all women in the study population received at least one ANC visit. Those who did not attend ANC were not asked about IFA receipt or consumption;

^b^ Early enrollment: 1^st^ trimester, Late enrollment: 2^nd^-3^rd^ trimester; ANC: Antenatal care; IFA: Iron and Folic Acid

A large proportion (80.5%) of HSCs that were serving these villages was out of IFA stock on the day of the survey. Just over half of HSCs observed Village Health Days, which serve as a monthly outreach to villages by health workers and provide an opportunity for community members to interact with health workers and receive basic services, advice, and preventative care[39]. Among these services are ANC check-ups, which should include IFA distribution[39, 40]. Many HSCs also did not receive oversight from the PHCs (65.4% did not receive written feedback, 45.6% did not receive a visit from a medical officer in the previous month) or village committees (80.4% of HSCs reported no VHSC present in the center’s coverage area). Half of included HSCs had 2 or more health workers (44.8%) and most reported receiving training on 4–8 of the topics surveyed (training topics: integrated skill development (Reproductive and Child Health—I), Vector Borne Disease Control Programme, Directly Observed Treatment Short course, Immunization, Intra Uterine Device Insertion, Integrated Management of Neonatal and Childhood Illnesses, Skilled Birth Attendant, and any other trainings)[41]. Only 13.6% of included HSCs received and utilized untied funds (annual funds provided for local needs as determined by the ANM) during the previous year. The majority of HSC buildings needed repair (69.5%) and most did not have any source of water available at the site (64.0%) (Table 5).

**Table 5:** Facility-level factors of study population by prevalence of iron and folic acid receipt and consumption^a^.

			*Received any IFA*	*Chi-Square*		*Consumed IFA for ≥90 days*	*Chi-Square*
		N	%	p-value	N	%	p-value
**Overall**		1012	37.4		890	23.8	
**HSC Distance from village** ^**b**^							
	*In village*	409	37.9	0.8364	373	25.5	0.0986
	*<5km*	492	37.1		421	23.6	
	*≥5km*	153	36.8		130	19.4	
**IFA stock available on day of survey**				0.0611			0.0304
	*Yes*	197	39.9		182	27.6	
	*No*	815	36.8		708	22.7	
**Factor 1: VHD & PHC/village monitoring**							
**Observation of any Village Health Day**				0.0609			0.005
	*No*	419	35.9		370	20.8	
	*Yes*	593	38.6		520	25.9	
**Written feedback from PHC**				0.0576			0.0423
	*No*	662	36.4		586	22.5	
	*Yes*	350	39.5		304	26.4	
**VHSC present in some villages in HSC area**				0.3213			0.1992
	*No*	814	37.0		716	23.2	
	*Yes*	198	38.9		174	26.1	
**Medical Officer visited HSC in previous month**				0.5713			0.5456
	*No*	461	37.9		402	24.4	
	*Yes*	551	37.0		488	23.3	
**Factor 2: Personnel characteristics**							
**Untied funds** ^**c**^				0.8213			0.9473
	*No*	874	37.3		774	23.8	
	*Yes*	138	37.8		116	23.6	
**HSC personnel**				0.4364			0.1464
	*0–1 worker*	559	36.9		487	25.0	
	*≥2 workers*	453	38.0		403	22.3	
**HSC training**				0.0966			0.7253
	*0–3 topics*	438	36.0		380	23.4	
	*4–8 topics*	574	38.5		510	24.0	
**Factor 3: Sub-Center infrastructure**							
**Present condition of existing building**				0.0082			0.0495
	*Needs repair*	703	36.1		611	25.0	
	*Satisfactory*	309	40.3		279	21.2	
**Water available at Sub-Center**				0.2328			0.0616
	*No*	364	36.3		319	21.7	
	*Yes*	648	38.1		571	25.1	

^a^ HSC counts are unweighted. Percentages and chi-square tests take into account weighting and clustering at the primary sample unit level.

^b^ HSCs which covered more than one village could be counted multiple times.

^c^ Received and utilized untied funds from previous financial year; HSC: Health Sub-Center; IFA: iron and folic acid; PHC: Primary Health Center; VHSC: Village Health and Sanitation Committee

### Individual Factors

When only individual demographic variables were considered, higher education, increased wealth, and lower birth order increased odds of both IFA receipt and consumption (Tables 6 and 7). Conversely, young age (<20 y), young age at marriage (<18 y), and non-Hindu religion were negatively associated with IFA receipt (Table 6). Women of scheduled castes or tribes had lower odds of consuming IFA for 90 or more days (Table 7). For both outcomes, including ANC variables attenuated most of the associations between the individual factors and outcomes. The addition of HSC factors did not meaningfully influence these relationships (Tables 6 and 7).

**Table 6:** Multilevel modeling of any iron and folic acid receipt during last pregnancy.

Parameter		Individual Factors	ANC Factors		HSC Factors	All Factors	All Factors + Interactions^a^
			Time	Quality			
		*OR (95% CI)*	*OR (95% CI)*	*OR (95% CI)*	*OR (95% CI)*	*OR (95% CI)*	*OR (95% CI)*
**Age**							
	*<20 y*	0.81 (0.66, 0.99)	0.89 (0.73, 1.10)	0.93 (0.76, 1.16)	0.81 (0.66, 0.99)	0.96 (0.77, 1.18)	0.95 (0.76, 1.17)
	*20–24 y*	1.03 (0.91, 1.18)	1.05 (0.92, 1.20)	1.08 (0.94, 1.25)	1.03 (0.91, 1.18)	1.09 (0.95, 1.25)	1.08 (0.94, 1.24)
	*>24 y*	1.00	1.00	1.00	1.00	1.00	1.00
**Age of marriage**							
	*<18 y*	1.00	1.00	1.00	1.00	1.00	1.00
	*≥18 y*	1.18 (1.04, 1.35)	1.20 (1.05, 1.37)	1.19 (1.04, 1.37)	1.18 (1.04, 1.35)	1.20 (1.04, 1.37)	1.21 (1.05, 1.39)
**Mother's education**							
	*None / Don't Know*	1.00	1.00	1.00	1.00	1.00	1.00
	*1–4 y*	1.18 (0.97, 1.44)	1.13 (0.93, 1.39)	1.00 (0.81, 1.23)	1.18 (0.97, 1.44)	1.00 (0.81, 1.23)	0.98 (0.79, 1.21)
	*5–8 y*	1.46 (1.25, 1.71)	1.44 (1.22, 1.63)	1.26 (1.07, 1.49)	1.46 (1.24, 1.71)	1.27 (1.07, 1.50)	1.26 (1.06, 1.49)
	≥9 y	2.42 (1.99, 2.95)	2.14 (1.75, 2.62)	1.69 (1.37, 2.08)	2.42 (1.99. 2.94)	1.67 (1.35, 2.05)	1.67 (1.35, 2.06)
**Gender composition of living children**							
	*No sons*	1.00 (0.88, 1.14)	0.97 (0.85, 1.11)	0.99 (0.86, 1.14)	1.00 (0.88, 1.14)	0.98 (0.85, 1.13)	0.97 (0.84, 1.12)
	*≥1 sons*	1.00	1.00	1.00	1.00	1.00	1.00
**Birth order of index pregnancy**							
	*1*	1.66 (1.39, 1.98)	1.41 (1.17, 1.68)	1.22 (1.01, 1.47)	1.66 (1.40, 1.99)	1.18 (0.98, 1.42)	1.18 (0.98, 1.42)
	*2*	1.36 (1.17, 1.58)	1.29 (1.10, 1.50)	1.16 (0.99, 1.36)	1.36 (1.17, 1.58)	1.15 (0.98, 1.35)	1.15 (0.98, 1.34)
	*≥3*	1.00	1.00	1.00	1.00	1.00	1.00
**Caste**							
	*Scheduled castes & tribes*	1.01 (0.88, 1.16)	1.06 (0.92, 1.21)	1.10 (0.96, 1.27)	1.01 (0.88, 1.16)	1.12 (0.97, 1.29)	1.13 (0.98, 1.30)
	*Others*	1.00	1.00	1.00	1.00	1.00	1.00
**Religion**							
	*Hindu*	1.00	1.00	1.00	1.00	1.00	1.00
	*Muslim & others*	0.74 (0.63, 0.88)	0.76 (0.64, 0.90)	0.77 (0.65, 0.92)	0.75 (0.64, 0.89)	0.79 (0.66, 0.94)	0.79 (0.66, 0.94)
**Wealth Index quintiles**							
	*Poorest*	1.00	1.00	1.00	1.00	1.00	1.00
	*Second*	1.23 (1.07, 1.41)	1.20 (1.05, 1.38)	1.12 (0.97, 1.29)	1.22 (1.07, 1.40)	1.11 (0.97, 1.28)	1.12 (0.97, 1.29)
	*Middle*	1.48 (1.25, 1.76)	1.34 (1.13, 1.59)	1.20 (1.43, 1.48)	1.48 (1.25, 1.75)	1.17 (0.98, 1.40)	1.16 (0.97, 1.39)
	*Fourth*	1.89 (1.51, 2.35)	1.68 (1.34, 2.10)	1.31 (1.04, 1.66)	1.87 (1.50, 2.33)	1.29 (1.02, 1.63)	1.30 (1.03, 1.64)
	*Richest*	2.48 (1.69, 3.63)	1.95 (1.31, 2.89)	1.30 (0.86, 1.95)	2.46 (1.68, 3.61)	1.23 (0.82, 1.86)	1.31 (0.87, 1.97)
**Husband's education**							
	*None / Don't know*	1.00	1.00	1.00	1.00	1.00	1.00
	*1–4 y*	1.29 (1.06, 1.57)	1.25 (1.03, 1.53)	1.20 (0.98, 1.47)	1.29 (1.06, 1.57)	1.19 (0.97, 1.45)	1.16 (0.95, 1.43)
	*5–8 y*	1.24 (1.06, 1.44)	1.21 (1.04, 1.42)	1.10 (0.94, 1.29)	1.24 (1.06, 1.44)	1.11 (0.94, 1.30)	1.09 (0.93, 1.28)
	*9–12 y*	1.38 (1.17, 1.61)	1.26 (1.07, 1.48)	1.10 (0.93, 1.30)	1.37 (1.17, 1.60)	1.08 (0.91, 1.27)	1.06 (0.90, 1.26)
	*>12 y*	1.62 (1.24, 2.12)	1.44 (1.09, 1.89)	1.19 (0.90, 1.59)	1.62 (1.23, 2.11)	1.17 (0.88, 1.56)	1.15 (0.86, 1.53)
**Antenatal care timing and frequency**							
	*Early enrollment* ^*b*^ *and* ≥*4 visits*		4.30 (3.56, 5.19)			1.85 (1.50, 2.28)	3.53 (2.44, 5.11)
	*Late enrollment* ^*b*^ *and* ≥*4 visits*		2.59 (2.61, 4.93)			1.75 (1.25, 2.45)	2.44 (1.69, 3.51)
	*Early enrollment* ^*b*^ *and <4 visits*		1.52 (1.35, 1.72)			1.27 (1.12, 1.43)	1.36 (1.19, 1.55)
	*Late enrollment* ^*b*^ *and <4 visits*		1.00			1.00	1.00
**ANC factors**							
	*Practice*			7.17 (5.89, 8.73)		5.56 (4.50, 6.87)	13.12 (9.51, 18.09)
	*Counseling*			1.90 (1.62, 2.21)		1.86 (1.59, 2.18)	2.61 (2.12, 3.21)
	Practice*Counseling interaction						0.37 (0.25, 0.56)
	Timing & frequency * Practice interaction						0.68 (0.56, 0.82)
**HSC factors**							
	*VHD & PHC/village monitoring*				1.13 (0.90, 1.40)	1.10 (0.88, 1.37)	1.11 (0.89, 1.38)
	*Personnel characteristics*				1.13 (0.87, 1.48)	1.03 (0.79, 1.35)	1.01 (0.77, 1.32)
	*Sub-Center infrastructure*				1.25 (0.93, 1.67)	1.21 (0.90, 1.62)	1.22 (0.91, 1.64)
**IFA stock available on day of survey**							
	*Yes*				1.09 (0.92, 1.29)	1.08 (0.91, 1.28)	1.09 (0.92, 1.29)
	*No*				1.00	1.00	1.00
**Distance to nearest HSC**							
	*In village*						
	*<5 km*				0.96 (0.83, 1.11)	0.94 (0.81, 1.09)	0.93 (0.80, 1.08)
	≥5 km				0.95 (0.78, 1.17)	0.90 (0.73, 1.11)	0.90 (0.73, 1.10)
**Community level random effect (SE)**		0.6636 (0.0442)	0.6441 (0.0457)	0.6250 (0.0469)	0.6585 (0.0443)	0.6180 (0.0473)	0.6259 (0.0473)
**AIC**		9421.26	9140.56	8712.50	9427.16	8683.59	8636.77

^a^ Significant interactions included ANC Practice * ANC Counseling and ANC Practice * ANC Timing & Frequency;

^*b*^ Early enrollment: 1^st^ trimester, Late enrollment: 2^nd^–3^rd^ trimester; AIC: Akaike Information Criterion; ANC: Antenatal Care; HSC: Health Sub-Center; PHC: Primary Health Center; SE: Standard Error

**Table 7:** Multilevel modeling of iron and folic acid consumption for ≥90 days during last pregnancy.

Parameter		Individual Factors	ANC Factors		HSC Factors	All Factors
			Time	Quality		
		*OR (95% CI)*	*OR (95% CI)*	*OR (95% CI)*	*OR (95% CI)*	*OR (95% CI)*
**Age**						
	*<20 y*	0.68 (0.47, 1.00)	0.81 (0.55, 1.21)	0.78 (0.53, 1.14)	0.67 (0.46, 0.98)	0.84 (0.57, 1.25)
	*20–24 y*	0.82 (0.64, 1.05)	0.84 (0.65, 1.09)	0.85 (0.66, 1.09)	0.81 (0.64, 1.04)	0.84 (0.65, 1.10)
	*>24 y*	1.00	1.00	1.00	1.00	1.00
**Age of marriage**						
	*<18 y*	1.00	1.00	1.00	1.00	1.00
	*≥18 y*	0.82 (0.65, 1.04)	0.82 (0.64, 1.06)	0.82 (0.64, 1.05)	0.81 (0.64, 1.02)	0.81 (0.63, 1.04)
**Mother's education**						
	*None / Don't know*	1.00	1.00	1.00	1.00	1.00
	*1–4 y*	1.13 (0.75, 1.69)	1.07 (0.7, 1.65)	1.05 (0.69, 1.60)	1.14 (0.76, 1.71)	1.05 (0.68, 1.62)
	*5–8 y*	1.39 (1.03, 1.88)	1.37 (1.00, 1.88)	1.26 (0.92, 1.71)	1.4 (1.04, 1.9)	1.31 (0.95, 1.80)
	≥9 y	2.16 (1.56, 2.98)	1.88 (1.34, 2.65)	1.84 (1.31, 2.57)	2.14 (1.55, 2.96)	1.75 (1.24, 2.48)
**Gender composition of living children**						
	*No sons*	1.06 (0.84, 1.34)	1.06 (0.82, 1.35)	1.08 (0.85, 1.37)	1.05 (0.83, 1.33)	1.05 (0.82, 1.35)
	*≥1 sons*	1.00	1.00	1.00	1.00	1.00
**Birth order of index pregnancy**						
	*1*	1.73 (1.27, 2.36)	1.31 (0.94, 1.82)	1.36 (0.99, 1.88)	1.76 (1.29, 2.41)	1.22 (0.88, 1.71)
	*2*	1.2 (0.91, 1.59)	1.08 (0.81, 1.44)	1.06 (0.8, 1.41)	1.23 (0.93, 1.62)	1.04 (0.77, 1.39)
	*≥3*	1.00	1.00	1.00	1.00	1.00
**Caste**						
	*Scheduled castes & tribes*	0.62 (0.46, 0.83)	0.68 (0.50, 0.92)	0.69 (0.51, 0.93)	0.62 (0.47, 0.83)	0.71 (0.53, 0.97)
	*Others*	1.00	1.00	1.00	1.00	1.00
**Religion**						
	*Hindu*	1.00	1.00	1.00	1.00	1.00
	*Muslim & others*	1.02 (0.74, 1.41)	1.01 (0.72, 1.42)	1.07 (0.76, 1.49)	1.02 (0.74, 1.41)	1.03 (0.73, 1.46)
**Wealth Index quintiles**						
	*Poorest*	1.00	1.00	1.00	1.00	1.00
	*Second*	1.3 (0.94, 1.79)	1.31 (0.94, 1.84)	1.16 (0.84, 1.61)	1.27 (0.92, 1.74)	1.18 (0.84, 1.66)
	*Middle*	1.23 (0.86, 1.77)	1.07 (0.74, 1.56)	1.01 (0.7, 1.46)	1.22 (0.85, 1.74)	0.96 (0.65, 1.4)
	*Fourth*	1.79 (1.2, 2.65)	1.59 (1.05, 2.41)	1.31 (0.87, 1.98)	1.75 (1.18, 2.60)	1.31 (0.86, 2.00)
	*Richest*	2.99 (1.78, 5.01)	2.62 (1.52, 4.54)	1.96 (1.15, 3.35)	2.95 (1.76, 4.95)	2.05 (1.17, 3.56)
**Husband's education**						
	*None / Don't know*	1.00	1.00	1.00	1.00	1.00
	*1–4 y*	1.06 (0.67, 1.68)	1.06 (0.66, 1.71)	0.97 (0.61, 1.56)	1.04 (0.66, 1.66)	0.98 (0.61, 1.60)
	*5–8 y*	1.26 (0.89, 1.78)	1.2 (0.84, 1.71)	1.11 (0.78, 1.59)	1.29 (0.91, 1.81)	1.13 (0.79, 1.63)
	*9–12 y*	1.64 (1.17, 2.29)	1.47 (1.03, 2.08)	1.36 (0.96, 1.92)	1.65 (1.18, 2.31)	1.32 (0.93, 1.89)
	*>12 y*	1.97 (1.27, 3.05)	1.65 (1.04, 2.61)	1.58 (1, 2.48)	1.98 (1.28, 3.07)	1.48 (0.93, 2.36)
**Antenatal care timing and frequency**						
	*Early enrollment* ^*a*^ *and* ≥*4 visits*		4.85 (3.66, 6.43)			3.4 (2.52, 4.59)
	*Late enrollment* ^*a*^ *and* ≥*4 visits*		4.27 (2.75, 6.62)			3.19 (2.03, 5.01)
	*Early enrollment* ^*a*^ *and <4 visits*		1.15 (0.89, 1.5)			1.05 (0.81, 1.37)
	*Late enrollment* ^*a*^ *and <4 visits*					
**ANC factors**						
	*Practice*			4.6 (3.35, 6.32)		2.62 (1.86, 3.71)
	*Counseling*			1.11 (0.86, 1.43)		1.08 (0.83, 1.4)
**HSC factors**						
	*VHD & PHC/village monitoring*				1.46 (1.03, 2.07)	1.43 (0.98, 2.09)
	*Personnel characteristics*				0.81 (0.53, 1.24)	0.87 (0.55, 1.38)
	*Sub-Center infrastructure*				0.73 (0.46, 1.17)	0.69 (0.42, 1.14)
**IFA stock available on day of survey**						
	*Yes*				1.33 (1.03, 1.71)	1.37 (1.04, 1.82)
	*No*				1.00	1.00
**Distance to nearest HSC**						
	*In village*				1.00	1.00
	*<5 km*				0.94 (0.74, 1.18)	0.95 (0.74, 1.22)
	≥5 km				0.72 (0.51, 1.02)	0.73 (0.51, 1.06)
**Community Level Random Effect**		0.702 (0.106)	0.794 (0.108)	0.733 (0.107)	0.682 (0.106)	0.781 (0.108)
**AIC**		2950	2780.37	2838	2946	2746.6

^a^ Early enrollment:

1^st^ trimester, Late enrollment: 2^nd^-3^rd^ trimester; AIC: Akaike Information Criterion; ANC: Antenatal care; HSC: Health Sub-Center; IFA: iron and folic acid; PHC: Primary Health Center

In our final multilevel model for IFA receipt, controlling for covariates and interactions, women were less likely to receive any IFA during their last pregnancy if they were Muslim or another religion (vs. Hindu) (OR: 0.79, 95% CI: 0.66, 0.94) and more likely to receive IFA if they were educated (5–8 y vs. none: OR: 1.26, 95% CI: 1.06, 1.49; ≥9 y vs. none: OR: 1.67, 95% CI: 1.35, 2.06), married as an adult (≥18 y vs. <18 y: OR: 1.21, 95% CI: 1.05, 1.39), and in the fourth richest quintile (Fourth vs. Poorest Quintile: OR: 1.30, 95% CI: 1.03, 1.64) (Table 6).

Fewer demographic variables were significantly associated with consumption of IFA for 90 or more days. Women were less likely to consume IFA for the recommended time frame if they were of a scheduled caste or tribe vs. others (OR: 0.71, 95% CI: 0.53, 0.97) and more likely to consume if they were in the richest wealth quintile vs. the poorest (OR: 2.05, 95% CI: 1.17, 3.56) or more educated (≥9 y vs. none: OR: 1.75, 95% CI: 1.24, 2.48) (Table 7).

### Antenatal Care Factors

ANC trimester of initiation and frequency were significantly positively associated with receipt of any IFA (early enrollment and ≥4 visits vs. late enrollment and <4 visits: OR: 3.53, 95% CI: 2.44, 5.11; late enrollment and ≥4 visits vs. late enrollment and <4 visits: OR: 2.44, 95% CI: 1.69, 3.51; early enrollment and <4 visits vs. late enrollment and <4 visits: OR: 1.36, 95% CI: 1.19, 1.55) (Table 6). Women had especially high odds of IFA receipt when they attended 4 or more ANC check-ups.

Women were much more likely to receive IFA if they received more services at their ANC appointments and when they received more counseling. ANC quality factors did show a significant interaction in this model (OR: 0.37, 95% CI: 0.25, 0.56). This indicates that as a woman receives increasing ANC services or counseling, her receipt of the other factor has less influence on whether or not she receives IFA. In addition, the ANC practice factor significantly interacted with the ANC timing and frequency variable as well (OR: 0.68, 95% CI: 0.56, 0.82)(Table 6). Examining this interaction further, we found that for women who attended <4 ANC visits, the ANC practice factor had a stronger impact on IFA receipt than women who attended 4 or more. Women who attended more ANC visits also were more likely to receive more ANC services, so there was less variation in this group.

Women who received any IFA were more likely to consume for 90 or more days if they attended 4 or more ANC appointments, regardless of the timing of their enrollment. Women were also more likely to consume IFA for 90 days if they received more antenatal care services (ANC services factor score 1-unit change: OR: 2.62, 95% CI: 1.86, 3.71) (Table 7). Receipt of counseling was not significantly associated with adequate IFA consumption. No significant interactions were found in this model.

### Health Sub-Center Factors

No HSC factors showed a significant relationship to women’s IFA receipt, including IFA supply and HSC proximity (Table 6).

Only the Village Health Day & PHC/Village Monitoring factor (OR: 1.46, 95% CI: 1.03, 2.07) and IFA supply (OR: 1.33, 95% CI: 1.03, 1.71) were significantly associated with IFA consumption when controlling for demographic variables. In the final model, women living in villages where the HSC had IFA in stock on the day of the survey were more likely to have consumed IFA for 90 or more days during their last pregnancy (OR: 1.37, 95% CI: 1.04, 1.82) (Table 7).

### Random Effects and Akaike Information Criterion

For each model presented, facility-level random effects are presented along with standard errors. In addition, AIC is reported to reflect overall fit of the model. For both outcomes of interest, the facility-level variation is significant, indicating that both IFA receipt and IFA consumption of 90 days or more varied by both facility-level factors in addition to individual characteristics of the women.

For IFA receipt, the addition of ANC variables (Timing and Frequency: 0.6641, Standard Error (SE): 0.0457; Quality: 0.6250, SE: 0.0469), HSC variables (0.6585, SE: 0.0443), and the cumulative models did appear to reduce the facility-level random effects slightly, meaning that these variables helped to explain some of the variation between PSUs. However, there are still significant differences between PSUs with regard to IFA receipt, as the random effects were still significant in our final model (0.6259, SE: 0.0473).

In addition, our final model shows the best fit using AIC values. In particular, ANC Quality factors seem to improve fit more so than ANC Timing and Frequency or HSC factors.

For IFA consumption for 90 days or more, facility-level variations did not decrease with addition of all factors to the model but rather increased slightly (Individual Model: 0.702; All Factors Model: 0.7805). Therefore in our analysis, our included ANC and HSC factors did not affect the variation between facility coverage areas. Unexplained variation still exists between these groups. However, AIC values do show that our final model fit the best out of those presented.

## Discussion

The results of this analysis demonstrate that individual and ANC factors are significantly associated with IFA receipt and consumption in this context. Surprisingly no HSC characteristics were associated with IFA receipt, although women covered by HSCs with IFA on the survey day did have higher odds of consuming IFA for 90 or more days.

### Individual Factors

Both IFA receipt and consumption improved with higher educational attainment and household wealth. This relationship has been found in many studies and shown to play a role in a variety of maternal health service utilization patterns as well as pregnancy outcomes[19, 33, 42]. Odds of IFA receipt were also significantly lower for those who married before 18 years of age and also for non-Hindus. Early marriage has been associated with inadequate maternal healthcare utilization[43, 44] and pregnancy complications[32] which may be due to lack of decision-making power within the household in health related matters[45]. In our sample, the majority of non-Hindu women were Muslim (99.5%). Several studies in India have shown that Muslim women utilize maternal healthcare less than Hindu women[44], including going to Accredited Social Health Activists (ASHAs) for antenatal and postnatal activities[46], institutional delivery (in Bihar)[47], skilled attendants at delivery[48, 49], and maternal and child IFA receipt[19]. One study suggested delivery care disparities in the Muslim community may be due to the practice of *purdah* (gender segregation)[48]. Women of scheduled castes or tribes were less likely to consume IFA for 90 or more days during their last pregnancy, though this was not a significant factor in IFA receipt. Pasricha et al’s analysis of NFHS-3 national data also found a non-significant relationship between scheduled caste/tribe and maternal IFA receipt. However, they did find children in scheduled caste households were less likely to receive IFA supplementation[19]. In a smaller study in Karnataka, mothers in scheduled castes were less likely to receive IFA[19]. Scheduled caste or tribal populations are also often of lower socio-economic status and socially excluded, leading to a lack of healthcare access, although this relationship existed when controlling for education and household wealth. All of these factors may impact both IFA receipt and adequate consumption[50]. It is interesting to note that these individual factors remained significant even when controlling for ANC services provided and counseling messages given. This may reflect a higher level of awareness to understand counseling messages or different levels of treatment given by health providers even when similar services were given[51].

### Antenatal Care Factors

Especially in low-resource settings, early and frequent ANC attendance may not translate into quality care or counseling[52]. Therefore, we constructed two factors to account for ANC quality: ANC service provision and ANC counseling topics covered. The ANC service factor proved to be significant in both successful receipt and consumption of IFA, even after controlling for timing of initiation and frequency of ANC. A study conducted in Lucknow, India found non-significant correlations between IFA consumption of ≥100 tablets and ANC timing or frequency. However, this study’s small sample size and method of recruitment (using local healthcare worker registers) contribute to limited statistical power and reduced ANC variation in the sample, diminishing the potential effect of ANC[22]. Our findings do corroborate the results of Pasricha et al. who also found ANC quality measures were significantly associated with IFA receipt in addition to ANC timing and frequency. Authors reported that women were more likely to have received antenatal IFA if they also received postnatal care or an antenatal blood test[19]. Our ANC measures included only components of antenatal care, which may be more useful when evaluating antenatal and postnatal care separately. In addition, the ANC service and ANC counseling factors may be applied to other outcomes during pregnancy as they provide a comprehensive assessment of high vs. low quality ANC instead of two specific practices.

For IFA receipt, we also found two significant interactions with ANC variables. The ANC practice and counseling interaction shows that as ANC services or counseling increase, the variation in the other factor has less influence on whether or not IFA is received. This indicates that at a certain point the overall quality of the ANC check-up is high enough that IFA is being received and slight fluctuations in services or counseling received do not influence receipt of IFA as much. In comparison, among women receiving very few services or minimal counseling, a slight improvement in quality in either domain, services or counseling, had a greater impact on IFA receipt.

A significant interaction was also found between ANC practice and ANC timing & frequency. Examining this further, we found that for women attending fewer visits, receiving more services had a greater impact on IFA receipt than for those attending four or more visits. This seems to show that with fewer visits, the quality of those visits becomes increasingly important with regard to IFA receipt. This may occur because of inconsistent IFA supply, where increased healthcare interaction increases the odds of IFA availability. This finding may also add to the current debate of the WHO antenatal care model, which encourages a reduced visit schedule[53]. Our data suggest that increasing the number of required ANC visits may increase opportunities for IFA access and counseling surrounding consumption, especially when service delivery is poor. More ANC visits would also increase opportunities for ANC service provision and repetition of counseling messages. However, under optimal conditions, fewer ANC visits would be needed. It is also possible that women who attended ANC four or more times may also have easier access to the HSC e.g. live near a major roadway. Individual and household beliefs surrounding healthcare utilization may also play a role. For example, women may have greater decision-making power within the household, increased autonomy, or have a greater say in their healthcare needs. Another explanation may be that these were women suffering from pregnancy complications, attended more appointments, and therefore more likely to receive IFA.

Among those women who did receive IFA, consumption for 90 days or more was significantly associated with both receipt of more ANC practices and greater number of ANC visits. Increased ANC frequency may positively impact IFA consumption through repeated health worker contact and follow up, reminding women to consume IFA and reinforcing counseling messages. Independently of repeated contacts, women who received more ANC services were also more likely to consume IFA for 90 or more days. This added correlation is often missed when including only ANC timing and frequency in models. The ANC counseling factor and early ANC enrollment with <4 appointments were not significantly associated with adequate IFA consumption. Though it was surprising that counseling did not impact IFA consumption, it should be noted that counseling specific to IFA was not asked about in the survey, which may have a more meaningful effect. In addition, the counseling variable was based on number of topics covered and did not take into account topic repetition or counseling quality. If <4 ANC visits were attended, initiating ANC in the first trimester did not increase the likelihood of consuming IFA for ≥90 days. Effective IFA counseling and health worker engagement have been shown in other studies to be important determinants of adequate IFA consumption. Women not understanding the correct dosage and forgetfulness were cited by Seck et al. as significant barriers to IFA adherence[20]. A qualitative study in Tamil Nadu also noted the motivation of health workers and perceived need by the community as important determinants of antenatal iron supplementation compliance[54]. These aspects of counseling effectiveness and community awareness were not assessed in our analysis.

### Health Sub-Center Factors

Studies show mixed results as to whether healthcare facility capacity is associated with earlier or more frequent ANC attendance. Some report that facility characteristics are associated with improved ANC utilization[49, 55, 56] though others cite non-significant relationships[57–59]. There is more consistency in the literature surrounding health facility access. Many studies do report travel time, distance, or health facility population coverage as significant factors in adequate ANC attendance[49, 55–57]. Haverkate et al.’s analysis of women’s hemoglobin status found that hemoglobin differences due to wealth quintiles were attenuated by increased number of health facilities[60]. In our analyses, HSC capacity, access, and supply factors were not associated with any IFA receipt, regardless of whether ANC variables were included in the model or not. HSC supply of IFA is dependent upon the supply available at the block, which depends on IFA from the district level. As such, HSC IFA supply may serve as a proxy for availability in the district. This lack of association may have been influenced by the fact that many women who received IFA did so through a private source (62.8%), as opposed to a public source (38.4%). Also, the study excluded women who did not attend ANC. It is possible that the existence of HSC facilities is associated with population-level access to IFA even if there is no association among women attending ANC.

HSC capacity did play a slightly larger role in adequate IFA consumption. When ANC factors were not included in the model, the HSC factor *Village Health Day and PHC/Village Monitoring* was significantly and positively associated with IFA consumption for 90 or more days. One goal of Village Health Days is to provide ANC to pregnant women at the village level, which includes IFA distribution[40, 61]. Therefore, it is encouraging that observation of Village Health Days and monitoring by the PHC and village was associated with increased IFA consumption. This relationship did become non-significant when ANC quality and frequency factors were included in the model. This may indicate that the Village Health Days’ role in increasing IFA consumption is through increased service provision and contact between the healthcare worker and beneficiary during ANC.

IFA consumption for 90 or more days was also significantly associated with HSC IFA supply on the day of the survey. This shows the importance of supply in providing adequate IFA to women, as this is clearly a requirement for consuming IFA for the recommended time period. IFA availability at the point of contact is required to receive additional IFA, which is often spread out over three visits, and to receive follow up IFA counseling. This agrees with other studies which show lack of IFA supply as a barrier to IFA adherence[23, 62, 63]. Seck et al. also suggested that the presence of IFA can affect not only receipt of IFA by beneficiaries but also quality of IFA counseling. They suggested that healthcare workers who did have IFA may have more effectively counseled on the rationale for taking IFA than those who did not[20].

### Strengths and Limitations

This study had several strengths. We utilized a state representative dataset of ever-married women in Bihar, India. This survey additionally linked healthcare facilities with the populations they serve, which made it possible to model health facility characteristics with relevant populations. In addition, we examined ANC quality as well as attendance and thus were able to measure the association of quality and timing/frequency to IFA receipt and consumption. We were also able to explore facility-level factors and their relationship to provision of IFA and consumption among beneficiaries.

Our analysis also had some limitations. All variables included in the model were based upon self-report by the women or facility workers except for HSC building condition (which was interpreted and reported by the interviewer). Women in our dataset were significantly different from the survey populations on several factors; most notably all had attended at least one ANC check-up. Therefore, our results are not generalizable to the women who did not access ANC. This was a significant proportion of the population (40%) who likely did not receive and/or consume IFA and the determinants of their ANC access may have differed from our study sample. Additionally, the women included in our analysis did not exclusively attend ANC from a government source. In fact, the majority received at least some ANC through the private sector (40.8% vs. 33.0% from the public sector) or obtained IFA through a private source. This may have reduced the influence of the public sector variables in our analysis as it was not the sole source of ANC received. In addition, it may have increased the correlation between wealth and services received as private ANC services are fee based. Our ANC counseling variable contained several important topics of ANC, though we did not have a variable specific to IFA counseling, which would have been more pertinent to our analysis. Finally, our IFA supply variable was only a measure of IFA supply availability on the survey day. However, this was the most complete variable for IFA supply and directly measured the supplement of interest. IFA supply availability was not measured at the block or district level.

### Implications

Overall, IFA receipt and consumption among pregnant women in rural Bihar are low and changes are needed to improve IFA delivery and compliance. Throughout Bihar, a consistent IFA supply is needed to provide women the opportunity to take IFA throughout pregnancy. Independent from ANC attendance and services provided, IFA supply was a significant factor in whether women consumed IFA for 90 days or more. Notably, public sector IFA supply remains a significant factor even though the population got IFA from private sources as well. It is also possible that the presence of IFA at antenatal care visits improved IFA counseling, which may have increased IFA compliance, as suggested by a study in Senegal[20]. Less than 20% of HSCs in our sample had IFA on the day of the DLHS-3 survey. The Government of Bihar has taken steps to improve the supply of IFA and other essential drugs with the recent formation of the Bihar Medical Services & Infrastructure Corporation Limited[64], which has the goal of streamlining drug procurement and logistics[65]. Hopefully these efforts will improve IFA supply in the region. It should also be noted that most women who did receive IFA received it from the private sector. Ensuring private providers and pharmacists have access to accurate information on IFA prophylaxis use during pregnancy may also aid in women’s understanding and appropriate use of iron supplements.

Our multilevel models also showed a significant amount of variation at the facility level, which was not accounted for in our analyses. In this data, most facilities covered one distinct village; therefore additional variation may have occurred at the facility or community level. Potential unmeasured community level factors include social norms surrounding healthcare utilization, social support, unmeasured access issues (e.g. flood prone communities), or women’s empowerment to make health decisions [49, 66]. Two multilevel analyses of maternal health utilization in India also found unexplained variance at the community and district levels for most outcomes, including ANC attendance. These studies focused on PHC characteristics at the district level and demographic variation at the community level[49, 58]. Jat et al. also found no influence of PHC factors on maternal health service outcomes and hypothesized that this may be due to private healthcare utilization[58]. In places with high use of private healthcare use, further research is needed as indicators of public sector healthcare quality only may not capture the full variation between healthcare and outcomes. Interventions which go beyond the individual and household level to target facility and community level factors will be critical in addressing issues of both IFA receipt and adequate consumption. IFA distribution and counseling through other means in addition to ANC such as the private sector or community groups has also been suggested in areas with poor ANC coverage or quality[67]. Further research could examine this unexplained variance which may give insight on additional strategies to increase IFA receipt and consumption in Bihar.
